# A fast Chebyshev exponential-integrator solver for stochastic PDEs on bounded domains with non-homogeneous Dirichlet boundary data

**DOI:** 10.1016/j.mex.2026.104094

**Published:** 2026-08-11

**Authors:** Ronobir Chandra Sarker, Md. Shahidul Islam, Atiqur Rahman

**Affiliations:** Department of Mathematics and Statistics, Bangladesh University of Business and Technology, Dhaka, Bangladesh

**Keywords:** Stochastic partial differential equations, Chebyshev collocation, Exponential integrators, Fourth-order exponential time-differencing Runge–Kutta (ETDRK4), Stochastic exponential Euler, Talbot contour, Ornstein–Uhlenbeck noise, Stochastic Allen–Cahn, Stochastic burgers, Bounded domains

## Abstract

We present a Chebyshev-collocation exponential-integrator scheme for parabolic stochastic partial differential equations (SPDEs) on a bounded interval with time-dependent non-homogeneous Dirichlet boundary data, where periodic-domain Fourier methods and standard deterministic solvers do not apply. The method combines Chebyshev–Gauss–Lobatto differentiation matrices for the spatial Laplacian; a stochastic exponential-Euler time step whose linear dynamics are propagated exactly via matrix φ-functions computed by Talbot-contour quadrature and whose stochastic convolution is sampled exactly in the operator eigenbasis; and a Clenshaw–Curtis noise-scaling rule that gives the correct continuum white-noise limit on the non-uniform grid. We validate the scheme for additive space–time white noise and temporally coloured Ornstein–Uhlenbeck noise, and benchmark it against semi-implicit Euler–Maruyama, a stabilized explicit Runge–Kutta–Chebyshev method (S-ROCK), and explicit Euler–Maruyama. On a linear benchmark with known stationary variance, the method reaches the Monte-Carlo floor (0.93% peak relative error over 120 realisations) and stays there across two decades of time step, while the competitors degrade. Temporal convergence is order one and spatial convergence is spectral. On the stochastic Allen–Cahn equation the scheme is 7.5 × faster than a fine-step semi-implicit Euler–Maruyama reference at matched accuracy, and on the stiff stochastic Burgers equation it remains stable across an 8 × wider time-step range than semi-implicit Euler–Maruyama.

## Graphical abstract

Submitted as a separate file (graphical_abstract.png). Image dimensions: 1328 × 531 pixels.

**Figure:**
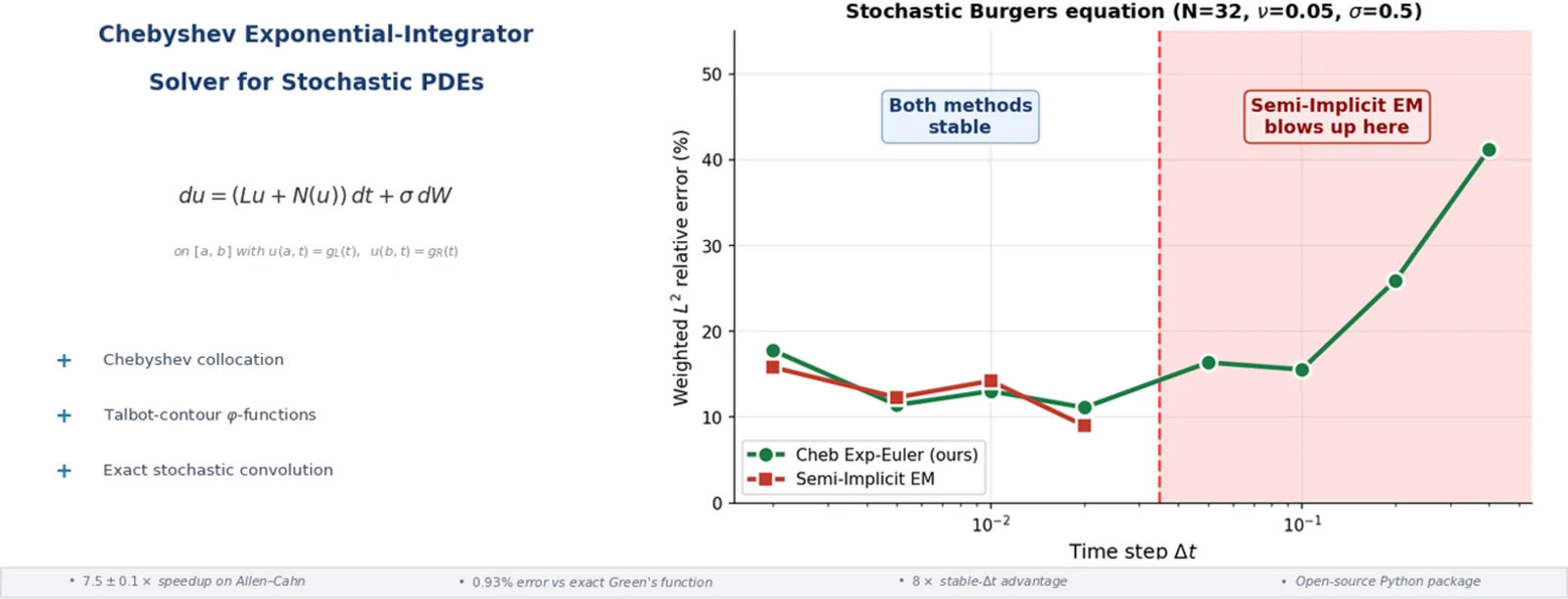

**Specifications table**

**Table:** 

**Subject area**	Mathematics and Statistics — Numerical Analysis
**More specific subject area**	Exponential integrators for stochastic partial differential equations; spectral methods on bounded domains
**Name of your method**	Chebyshev exponential-integrator SPDE solver (Cheb-ExpInt-SPDE)
**Name and reference of original method**	Cox & Matthews (2002) ETDRK4 [1]; Kassam & Trefethen (2005) [2]; Trefethen, Weideman & Schmelzer (2006) Talbot contour [3]; Lord & Tambue (2013) stochastic exponential Euler [4]; Waldvogel (2006) Clenshaw–Curtis weights [5]; Abdulle & Cirilli (2008) S-ROCK [6].
**Resource availability**	Source code (Python 3.9+, MIT license): https://github.com/Ronobir1sarker/cheb-spde Required dependencies: NumPy, SciPy, Matplotlib (https://pypi.org)

## Background

Parabolic stochastic PDEs (SPDEs) on bounded intervals arise throughout physics, biology, and engineering: stochastic Allen–Cahn for phase-field fronts, stochastic Burgers for turbulence and surface growth, stochastic reaction–diffusion for noise-driven pattern formation. The canonical form is

(1) $$du=\left(Lu+N\left(u\right)\right)\;dt+dW_{\text{noise}},x\in \left[a,b\right],u\left(a,t\right)=g_{L}\left(t\right),\;u\left(b,t\right)=g_{R}\left(t\right),$$

with $L=\partial_{x}^{2}$ the Laplacian, N(u) a pointwise nonlinearity, and $dW_{\text{noise}}$ either space–time white noise of intensity σ or an Ornstein–Uhlenbeck (OU) field with correlation time τ and strength σ. The two dominant numerical approaches are stabilised Runge–Kutta–Chebyshev schemes (S-ROCK [6] and its successors) and semi-implicit Euler–Maruyama (SI-EM) on a finite-difference or Fourier grid. Both are strong-order 1 methods with well-understood stability: S-ROCK has an explicit stability polynomial that widens the stable interval along the negative real axis but still restricts Δt proportional to 1/ρ(L); SI-EM is unconditionally linearly stable but the implicit solve damps the stochastic increments incorrectly at large Δt, degrading the statistics.

For bounded domains with non-periodic, non-homogeneous boundary data, the natural spatial discretization is Chebyshev collocation, whose differentiation matrices give spectral accuracy for smooth fields. Combining Chebyshev collocation with explicit Euler–Maruyama in time forces extremely small Δt because the Chebyshev Laplacian has spectral radius proportional to $N^{4}$. S-ROCK can mitigate this but still scales as $\Delta t\propto N^{-2}$ at matched stability. Semi-implicit EM allows larger Δt but pays in statistical bias.

We describe here a stochastic exponential-Euler scheme in the sense of Lord & Tambue [4] and Jentzen & Kloeden [7], adapted to the dense, non-normal Chebyshev operator that arises on a bounded Dirichlet domain. The linear part is propagated exactly by matrix φ-functions evaluated via the Trefethen–Weideman–Schmelzer Talbot contour [3]; the stochastic-convolution integral is computed in closed form in the eigenbasis of $L_{\text{int}}$ and sampled exactly by a Cholesky factor. The ingredient specific to the Chebyshev grid is the noise-scaling rule: on the non-uniform Chebyshev–Gauss–Lobatto grid the discrete white-noise increment is scaled by the inverse square root of the Clenshaw–Curtis quadrature weight at each node [5] rather than by a uniform $1/\sqrt{\Delta x}$, which is required to recover the correct continuum white-noise limit.

The result is a method whose per-step cost is dominated by two $O(N^{2})$ matrix–vector multiplications (the same as the deterministic fourth-order exponential time-differencing Runge–Kutta scheme, ETDRK4), whose setup cost is $O(N^{3})$ once per $\left(L_{\text{int}},\Delta t\right)$ pair, and whose stable time step is limited by accuracy rather than by the spectral radius of L. On stochastic Allen–Cahn and stochastic Burgers we demonstrate wall-clock and stable-step advantages of roughly one order of magnitude over semi-implicit Euler–Maruyama at matched statistical accuracy.

**Novelty and contribution.** The individual components used here are all established: Chebyshev collocation [8], exponential integrators and ETDRK4 [1,2], Talbot-contour evaluation of matrix functions [3], stochastic exponential Euler [4,7], and Clenshaw–Curtis quadrature [5]. We do not claim any of these as new. The contribution of this note is their *correct combination for a non-periodic, non-homogeneous Dirichlet problem discretised by a dense, strongly non-normal Chebyshev operator*, together with three specific points that this setting forces: (i) a Clenshaw–Curtis noise scaling that reproduces the exact stationary variance on the clustered grid, where uniform or finite-difference-style scalings do not; (ii) evaluation of the stochastic-convolution covariance in closed form in the eigenbasis of the *non-normal* interior Laplacian, for which the scalar Kassam–Trefethen contour recipe [2] is not a matrix function in the sense of Higham [9]; and (iii) the resulting decoupling of the stable time step from the $O(N^{4})$ spectral radius of the Chebyshev Laplacian. For broader context on exponential integrators we refer the reader to the review of Hochbruck & Ostermann [10].

## Method details

The method has four components, each implemented as an independent Python module (see Resource availability). We describe them in implementation order.

### Chebyshev–Gauss–Lobatto grid and differentiation matrices

We use Chebyshev points of the second kind on the physical interval [a,b]:

(2) $$x_{k}=\frac{a+b}{2}+\frac{b-a}{2}\;\text{cos}\left(\frac{k\pi }{N}\right),k=0,1,\ldots ,N,$$

giving N+1 nodes, clustered near the endpoints. Let $D\in R^{\left(N+1)\;\times \;(N+1\right)}$ be the first-derivative differentiation matrix built from the barycentric weights $w_{j}={\left(-1\right)}^{j}$ (with $w_{0}$ and $w_{N}$ halved), and $L=D^{2}$. The diagonal of D is fixed by the negative-sum trick $D_{ii}=-\sum_{j\neq i}D_{ij}$, which guarantees exact differentiation of constants. We refer the reader to Trefethen [8] for the explicit formulas.

**Implementation note.** The cosine map is monotonically decreasing, so $x_{0}=b$ and $x_{N}=a$. This reversed ordering is standard in spectral-methods implementations and is preserved throughout the code; no reordering is performed.

### Boundary lifting and complex-step time derivative

Given time-dependent Dirichlet boundary data $u\left(a,t\right)=g_{L}\left(t\right)$ and $u\left(b,t\right)=g_{R}\left(t\right)$, define the linear lift

(3) $$l\left(x,t\right)=\frac{b-x}{b-a}\;g_{L}\left(t\right)+\frac{x-a}{b-a}\;g_{R}\left(t\right),$$

and write u=v+l. Then v satisfies homogeneous Dirichlet boundary conditions v(a,t)=v(b,t)=0, and the evolution equation for v is

(4) $$dv=\left(L_{\text{int}}v+\tilde{F}\left(v,t\right)\right)\;dt+dW_{\text{noise}}\left(t\right),$$

where $L_{\text{int}}=L_{1:N-1,\;1:N-1}$ is the principal submatrix of L acting on interior nodes, and $\tilde{F}\left(v,t\right)$ collects the lifted nonlinearity, the boundary columns of L applied to the prescribed boundary values, and the time derivative ∂l/∂t. The last quantity is evaluated by complex-step differentiation,

(5) $$g_{L}^{{}^{\prime }}\left(t\right)\approx \frac{\text{Im}\left[g_{L}\left(t+i\varepsilon \right)\right]}{\varepsilon },\varepsilon =10^{-20},$$

which is accurate to round-off for any analytic $g_{L}\left(\cdot \right)$ and avoids the subtractive cancellation of finite differences [11,12]. Because the complex-step estimate has no cancellation error, its truncation error is $O(\varepsilon^{2})$ and ε may be taken far below the finite-difference optimum $\sqrt{\varepsilon_{\text{mach}}}\approx 10^{-8}$; the value $\varepsilon =10^{-20}$ is the standard choice [12] that keeps the imaginary part safely above underflow while making the truncation error negligible. The boundary-data callables must therefore accept complex arguments — a trivial requirement in Python/NumPy.

### **Matrix**φ**-functions by Talbot-contourquadrature**

The exponential-integrator update requires $\varphi_{0}\left(hL_{\text{int}}\right)=\text{exp}\left(hL_{\text{int}}\right)$ and

(6) $$\varphi_{1}\left(z\right)=\frac{e^{z}-1}{z},$$

as a matrix function of $A=L_{\text{int}}$. Kassam & Trefethen [2] proposed a scalar contour average around each eigenvalue to cancel catastrophic cancellation for small |z|. This works well when A is diagonal or nearly normal (e.g. a Fourier Laplacian on a periodic domain), but $L_{\text{int}}$ on a Chebyshev grid is full, non-symmetric, and strongly non-normal, and the scalar recipe is not a matrix function in the sense of Higham [9].

We evaluate $\varphi_{k}\left(hL_{\text{int}}\right)$ directly by a Trefethen–Weideman–Schmelzer Talbot contour [3]:

(7) $$\varphi_{k}\left(hA\right)=\frac{1}{2\pi i}\oint e^{hs}\;s^{-k}\;{\left(sI-A\right)}^{-1}\;ds,$$

discretised by a midpoint rule on the optimal-parabola contour $s\left(\theta \right)=\left(n_{q}/h\right)\;\psi \left(\theta \right)$, with

(8) $$\psi \left(\theta \right)=A\;\frac{\theta }{\text{tan}\left(B\;\theta \right)}-C+i\;D\;\theta ,$$

and fixed contour constants (A,B,C,D)≈(0.5017,0.6407,0.6122,0.2645). These are not free parameters: they are the values that minimise the estimated midpoint-rule quadrature error on the cotangent (Talbot) contour, tabulated by Trefethen, Weideman & Schmelzer [3] and Weideman & Trefethen [13], and they depend only on the contour family, not on the operator A. Each contour node requires one dense LU solve of (sI−A); with $n_{q}=32$–64 nodes this delivers double-precision accuracy on the spectra we encounter. The quadrature is performed once per $\left(L_{\text{int}},\Delta t\right)$ pair at setup and amortised over the entire time integration.

### Clenshaw–Curtis noise scaling on the non-uniform grid

A space–time white-noise increment dW(x,t) has covariance 〈dW(x)dW(y)〉=dtδ(x−y). On a uniform grid with spacing Δx the discrete analogue is $\delta W_{i}∼N\left(0,dt/\Delta x\right)$ because $\delta_{ij}/\Delta x\to \delta \left(x-y\right)$ in the continuum limit. On a non-uniform grid the same limit requires scaling by the inverse of the spatial integration weight at each node, so that

(9) $$\delta W_{i}∼N\left(0,dt/w_{i}\right),$$

where $w_{i}$ is the weight at node $x_{i}$ in a quadrature rule exact for smooth functions. For a Chebyshev–Gauss–Lobatto grid the natural choice is the Clenshaw–Curtis rule; we compute the weights by the Waldvogel [5] DCT-based formula. On [a,b] the weights are strictly positive, sum to b−a, and integrate polynomials of degree ≤N−1 to round-off. For N=8,16,32, the maximum error in the quadrature of $x^{k}$ over k=0,…,N drops from $10^{-4}$ (N=8) to $5\;\times 10^{-13}$ (N=32).

**Common pitfall.** A tempting alternative is to scale the noise uniformly by $1/\sqrt{\Delta x}$ with Δx≈π(b−a)/(2N) (a grid-average) or by $1/\sqrt{\Delta x_{i}}$ using finite-difference-style local spacings. Neither reproduces the continuum white-noise covariance on the clustered grid: relative to the quadrature-consistent weighting they over-weight the densely clustered boundary nodes and under-weight the interior. Among quadrature-consistent scalings on the Chebyshev–Gauss–Lobatto grid, the Clenshaw–Curtis rule is the natural choice, and it is the one that reproduces the exact stationary variance to the Monte-Carlo floor on the linear benchmark, whereas the two scalings above do not (see the linear-SPDE validation below).

### Stochastic exponential Euler on the interior grid

One step of size h from $t_{n}$ to $t_{n+1}$ advances the interior unknown $v_{\text{int}}\in R^{N-1}$ as

(10) $$v_{n+1}=\varphi_{0}\left(hL_{\text{int}}\right)\;v_{n}+h\;\varphi_{1}\left(hL_{\text{int}}\right)\;\tilde{F}\left(v_{n},t_{n}\right)+\xi_{n},$$

where $\xi_{n}=\int_{t_{n}}^{t_{n+1}}e^{\left(t_{n+1}-s\right)L_{\text{int}}}\;dW_{\text{int}}\left(s\right)$ is the exact stochastic convolution over one step. By the Itô isometry for Hilbert-space-valued Wiener processes [14,15], $\xi_{n}$ is Gaussian with mean zero and covariance

(11) $$C=\sigma^{2}\int_{0}^{h}e^{sL_{\text{int}}}\;W^{-1}\;e^{sL_{\text{int}}^{⊤}}\;ds,$$

where $W=\text{diag}(w_{1},\ldots ,w_{N-1})$ is the diagonal of interior Clenshaw–Curtis weights. Because $L_{\text{int}}$ is dense and non-normal, we diagonalise it once as $L_{\text{int}}=V\Lambda V^{-1}$ and evaluate C in the eigenbasis: with $\tilde{S}=V^{-1}W^{-1}{\left(V^{*}\right)}^{-1}$,

(12) $${\tilde{C}}_{ij}=\sigma^{2}\;{\tilde{S}}_{ij}\;\frac{e^{\left(\lambda_{i}+{\overset{‾}{\lambda }}_{j}\right)h}-1}{\lambda_{i}+{\overset{‾}{\lambda }}_{j}},$$

with the limit h as $(\lambda_{i}+{\overset{‾}{\lambda }}_{j})\to 0$. In the implementation this removable singularity is detected entrywise by the threshold test $|\lambda_{i}+{\overset{‾}{\lambda }}_{j}|<10^{-14}$ (routine _build_white_noise_factor, called from prepare_step, in spde_solver.py); flagged entries are assigned the analytic limit h directly, via a vectorised numpy.where, so no division by a near-zero denominator ever occurs. Mapping back to physical space gives $C=V\tilde{C}V^{*}$, which is real and symmetric positive semidefinite by construction; we symmetrise to kill floating-point imaginary parts and take Cholesky with a small adaptive jitter if the smallest eigenvalue is at round-off. The per-step cost of sampling $\xi_{n}$ is one Cholesky-factor multiplication, i.e. $O(N^{2})$.

For Ornstein–Uhlenbeck noise η(x,t) with correlation time τ and strength σ, we treat η as a separate process integrated exactly by

(13) $$\eta_{n+1,i}=\alpha \;\eta_{n,i}+\sigma \sqrt{\frac{1-\alpha^{2}}{w_{i}}}\;z_{n,i},\alpha =e^{-h/\tau },z_{n,i}∼N\left(0,1\right),$$

which reproduces the stationary variance $\sigma^{2}/w_{i}$ and the continuum autocorrelation $e^{-|s|/\tau }$ exactly. During the SPDE time step, $\sigma \cdot \eta (\cdot ,t_{n})$ enters as an additive drift in $\tilde{F}$, giving weak order 1 in h.

### Pseudocode

**Table:** 

**Algorithm 1: Chebyshev exponential-Euler SPDE solver**
**Input:** Grid size N; domain [a, b]; boundary functions g_L(t), g_R(t);
nonlinearity N(·); noise intensity σ; final time T; time step Δt
**Output:** Approximate solution u(·, T) at the Chebyshev nodes
**Stage 1: Precomputation (executed once)**
Build Chebyshev–Gauss–Lobatto nodes {x_j}_(j = 0..N) and second-order
differentiation matrix L on [a, b]
Restrict to interior nodes: L_int ← L_(1:N − 1, 1:N − 1)
Compute Clenshaw–Curtis weights {w_j}_(j = 0..N)
Compute matrix φ-functions φ_0(Δt L_int) and φ_1(Δt L_int) via
Talbot-contour quadrature with n_q = 64 contour points
Compute Cholesky factor C of the stochastic-convolution covariance Σ_Δt
**Stage 2: Time stepping**
v ← u_0(x_int) − ũ(x_int, 0) *// shift to homogeneous boundary*
**for n** **=** **0, 1, …, ⌈T/Δt⌉ − 1 do**
t ← n · Δt
F ← F̃(v, t) *// nonlinearity + complex-step time*
*// derivative of lift*
Sample η∼*N* (0, I_(N − 1))
v ← φ_0(Δt L_int) v + Δt φ_1(Δt L_int) F + σ C η
**end**
return v + ũ(x_int, T) *// add lift back to recover u*

## Method validation

We validate the method on a deliberately chosen progression of problems, each isolating a distinct property of the scheme: a *deterministic* limit that probes spatial (spectral) accuracy of the propagator; a *linear SPDE* with a closed-form Green’s-function stationary variance, which is the sharpest available check of the noise scaling and the stochastic convolution; a *stochastic Allen–Cahn* equation that adds a bistable pointwise nonlinearity and noise-driven escape; a *stochastic Burgers* equation that adds stiff, convection-dominated, chaotic dynamics and therefore stresses stability at large Δt; and *Ornstein–Uhlenbeck-driven* problems that test temporally coloured noise (correct autocorrelation and stationary variance). We also report a systematic spatial and temporal convergence study. Numerical values quoted below are reproduced by the scripts in the accompanying repository; the figures are the raw outputs of those scripts. All timings and hardware are specified in the *Reproducibility and performance protocol* below.

Throughout this section, “Monte-Carlo (MC) noise” or the “MC floor” refers to the statistical standard error of an ensemble estimator: for a quantity estimated as the mean of M independent realisations with across-realisation sample standard deviation s, the standard error is $\text{SE}=s/\sqrt{M}$, and a statement that an observed deviation is “within MC noise” means that it is smaller than ≈2SE (a 95% confidence interval, mean±1.96SE). Ensemble sizes range from M=12 per ensemble (repeated over four independent ensembles) in the benchmark scans to M=120 in the stationary-variance validation; the corresponding SE is reported alongside every accuracy claim, so the reader can judge each comparison against its own statistical resolution.

### Deterministic limit (spectral convergence)

Setting σ=0 and N(u)=0 reduces the solver to the exact linear propagator $v_{n+1}=\text{exp}\left(hL_{\text{int}}\right)v_{n}$. On the heat equation $u_{t}=u_{xx}$, u(±1,t)=0, with Fourier-mode initial condition $u_{0}\left(x\right)=\text{sin}\left(\pi (x+1)/2\right)$ (an eigenfunction of the Dirichlet Laplacian with eigenvalue $-{\left(\pi /2\right)}^{2}$), the $L^{\infty }$ error between the solver output and the exact solution $u\left(x,t\right)=e^{-{\left(\pi /2\right)}^{2}t}\text{sin}\left(\pi (x+1)/2\right)$ is:

**Table:** 

N	$L^{\infty }$**error at**T=0.3
8	$4.7\;\times 10^{-8}$
12	$4.2\;\times 10^{-13}$
16	$1.4\;\times 10^{-15}$
24	$1.6\;\times 10^{-14}$
32	$4.7\;\times 10^{-14}$
48	$4.7\;\times 10^{-14}$

The error drops to the round-off floor at N=16 and grows mildly thereafter from accumulated floating-point error in the Talbot quadrature, exactly as expected for a spectrally-accurate exact propagator.

### Linear SPDE: exact stationary variance

**Fig. 1:**
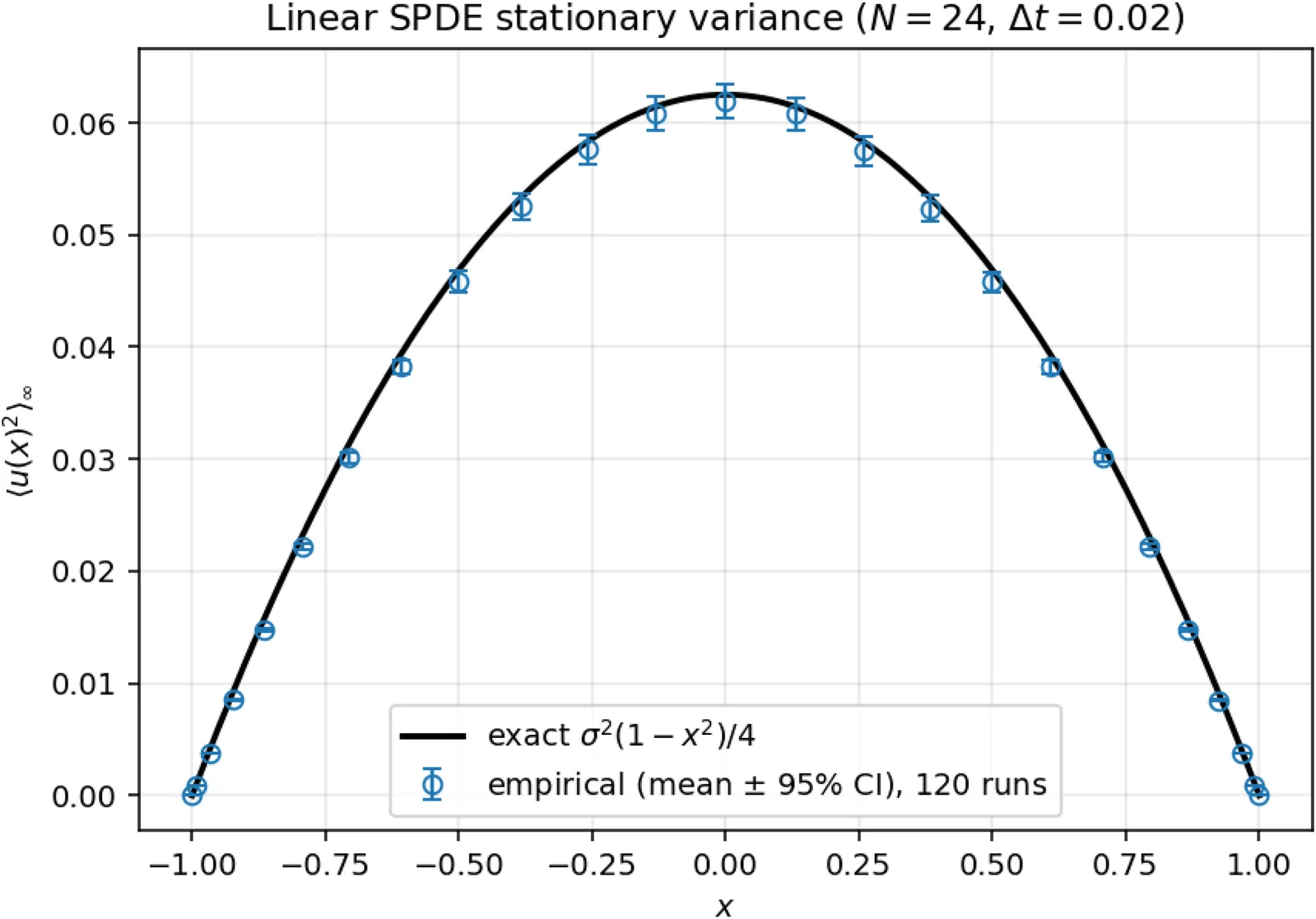
Stationary variance of the linear SPDE $du=u_{xx}\;dt+\sigma \;dW$ on [−1,1] with σ=0.5. Solid curve: exact Green’s-function prediction $\sigma^{2}\left(1-x^{2}\right)/4$. Markers: empirical variance from 120 solver runs (N=24, Δt=0.02, time-averaged over t∈[5,40]) shown as ensemble mean ± 95% confidence interval (mean±1.96SE). Peak relative error: 0.93% (within one standard error of the Monte-Carlo noise).

For $du=u_{xx}\;dt+\sigma \;dW$ on [−1,1] with homogeneous Dirichlet data, the stationary variance has the closed form

(14) $${\langle u{\left(x\right)}^{2}\rangle }_{\infty }=\frac{\sigma^{2}}{2}\;G\left(x,x\right)=\frac{\sigma^{2}\left(1-x^{2}\right)}{4},$$

where G(x,y)=(1−max(x,y))(1+min(x,y))/2 is the Green’s function of $-d^{2}/dx^{2}$ with Dirichlet boundary conditions on [−1,1]. Equation (14) is the sharpest available check for an SPDE solver on a bounded interval: if the noise scaling or the stochastic convolution is wrong, the empirical variance misses the exact curve by an amount far exceeding Monte-Carlo error. With N=24, σ=0.5, Δt=0.02, and 120 realisations of T=40 time-averaged after a $T_{\text{burn}}=5$ transient, we obtain the comparison in Fig. 1, where the empirical variance is shown as an ensemble mean with 95% confidence intervals. The relative error at the peak (x=0) is 0.93%, within one standard error of the Monte-Carlo noise (1.23%), confirming the absence of bias.

### Linear benchmark vs. three established integrators

We benchmark accuracy against three established integrators implemented on the identical Chebyshev/Dirichlet grid, so that only the time integrator differs: (i) semi-implicit Euler–Maruyama (SI-EM), the standard unconditionally (linearly) stable implicit method; (ii) a stabilized explicit Runge–Kutta–Chebyshev method (S-ROCK) in the sense of Abdulle & Cirilli [6], with the number of stages chosen so the extended real-axis stability interval covers $\Delta t\;\rho (L_{\text{int}})$; and (iii) explicit Euler–Maruyama, the naive explicit baseline. The error metric is the maximum relative error in the stationary variance over the middle half of the interval (avoiding tiny-variance boundary nodes), reported as the mean ± 1 s.d. over four independent ensembles of 12 realisations each.

**Fig. 2:**
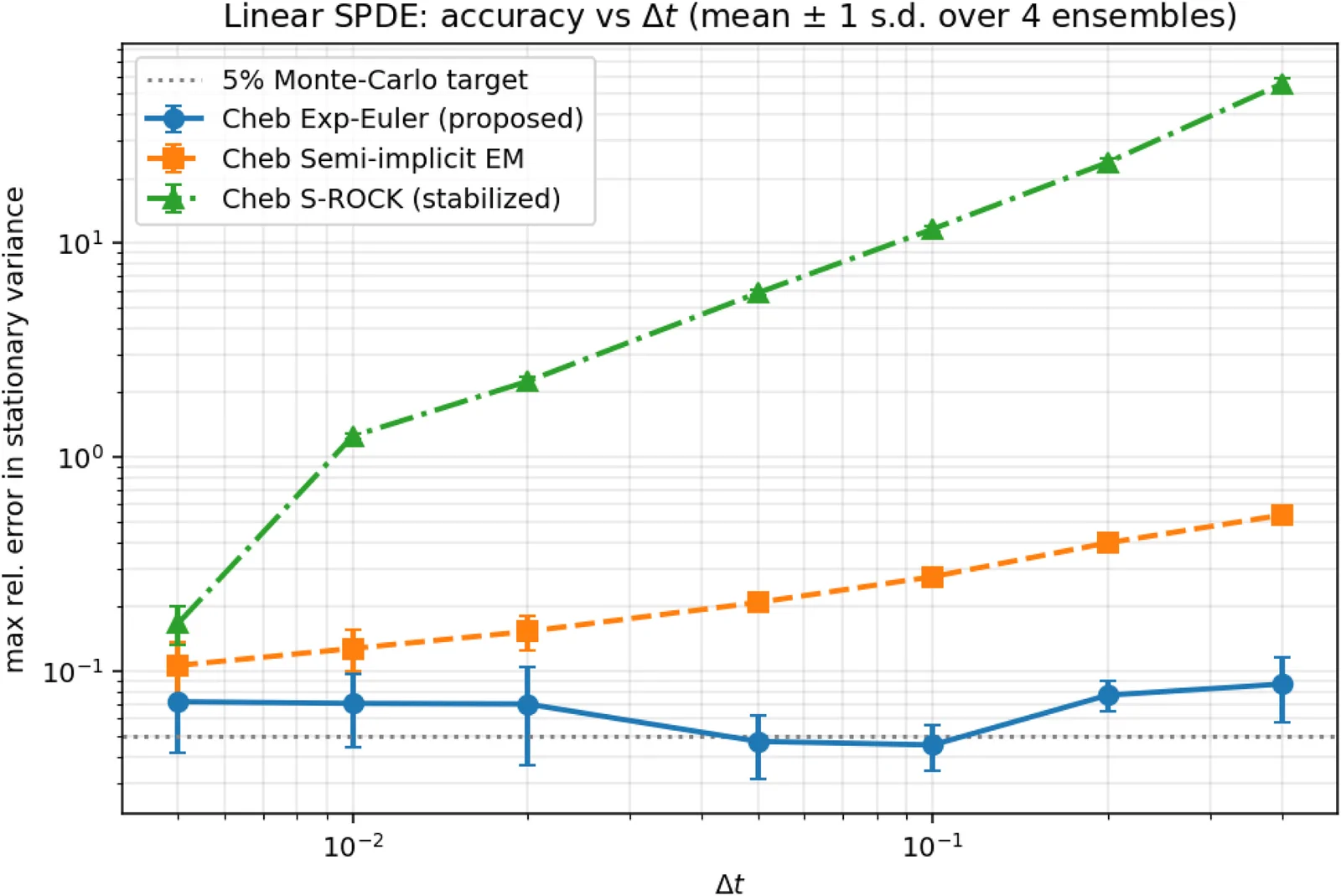
Linear SPDE benchmark, N=24, σ=0.5. Maximum relative error in the stationary variance versus Δt for the proposed Chebyshev exponential-Euler (circles), semi-implicit Euler–Maruyama (squares), and stabilized-explicit S-ROCK (triangles), all on the same grid. Markers are ensemble means and error bars are ± 1 standard deviation over four independent ensembles. Dotted line: 5% Monte-Carlo target. The proposed method stays at the Monte-Carlo floor across the whole range; SI-EM over-damps and S-ROCK over-injects at large Δt.

Figure 2 shows a clear separation. Our exponential-Euler stays at the Monte-Carlo floor (≈5%) across two decades of Δt. SI-EM degrades monotonically from 11% at Δt=0.005 to 54% at Δt=0.4 because the implicit solve over-damps the stochastic increments at large Δt. S-ROCK degrades far faster, from 17% to over 5000%, for the opposite reason: its strongly damped stabilized drift, combined with a naively added additive white-noise increment, over-injects variance into the stiff high modes when $\Delta t\;\rho (L_{\text{int}})\gg 1$. (Explicit Euler–Maruyama is omitted from the plot because it is unstable for every Δt shown; on this grid it requires Δt<∼2/ρ(Lint)≈1.3×10−4 for N=24.) The two competing methods thus fail in opposite directions — one over-damping, the other over-injecting — which is precisely what an exact linear propagator together with the exact Itô-isometry covariance avoids.

### Stochastic Allen–Cahn (nonlinear, bistable)

**Fig. 3:**
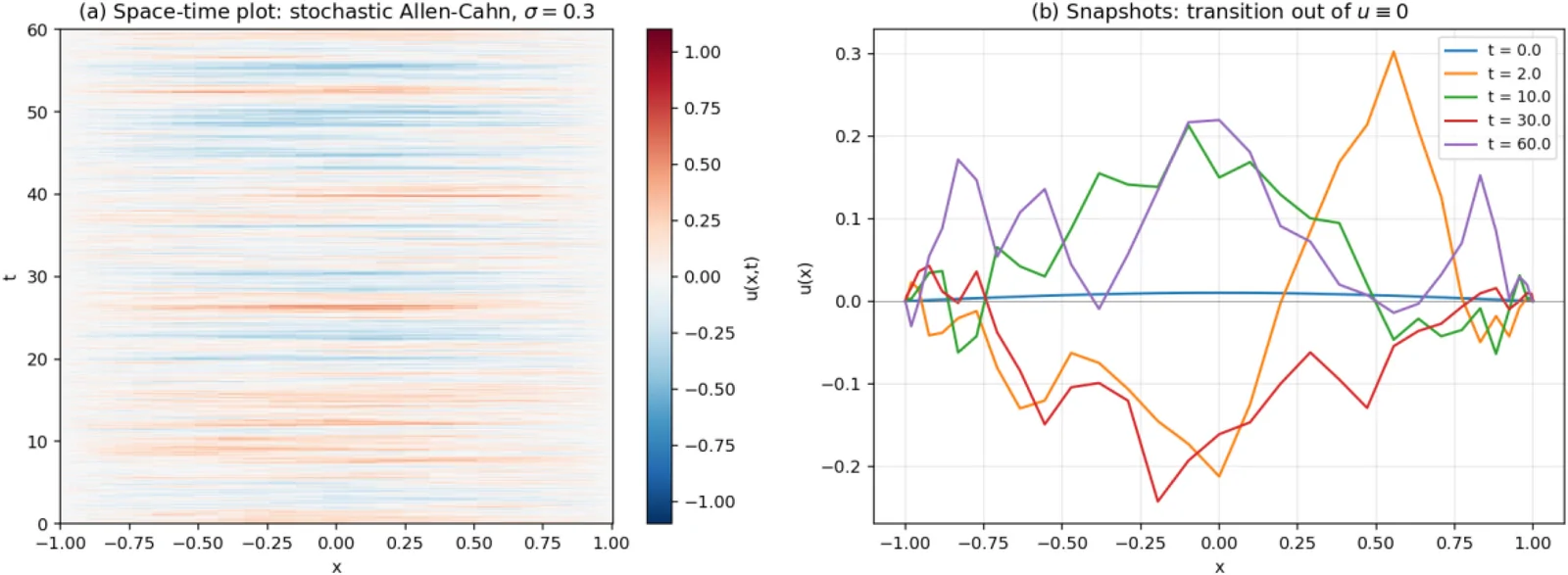
Stochastic Allen–Cahn dynamics on [−1,1] with σ=0.3, N=32, Δt=0.02. (a) Space–time plot showing noise-driven exit from the unstable equilibrium u≡0 and motion toward one of the bistable branches. (b) Spatial snapshots at t=0,2,10,30,60.

**Fig. 4:**
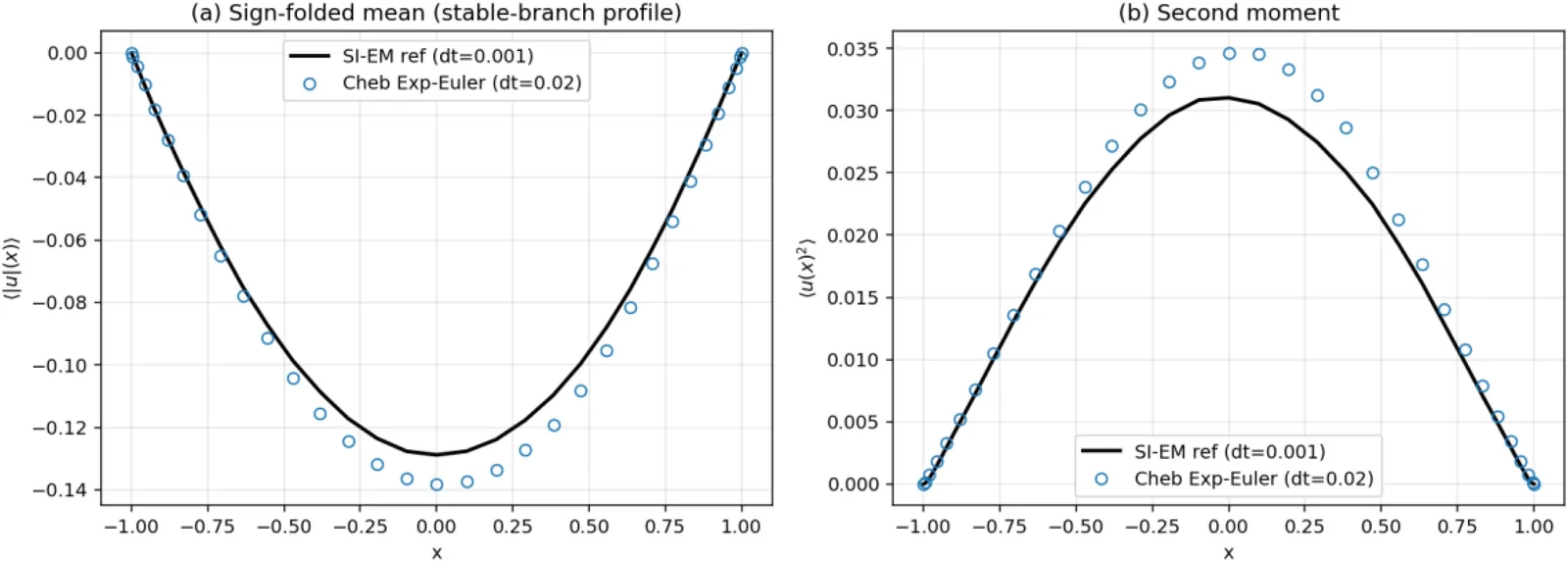
Stochastic Allen–Cahn ensemble statistics at T=20, 24 realisations. (a) Sign-folded first moment 〈|u|(x)〉. (b) Second moment $\langle u^{2}\left(x\right)\rangle$. Solid black: semi-implicit EM reference at $\Delta t=10^{-3}$. Open circles: our exponential-Euler at $\Delta t=2\;\times 10^{-2}$ (7.5±0.1× speedup). Agreement is within Monte-Carlo noise.

This case adds a bistable pointwise nonlinearity and tests accuracy of the nonlinear (exponential-Euler) update in a regime of noise-driven escape. For $du=(u_{xx}+u-u^{3})\;dt+\sigma \;dW$ on [−1,1], σ=0.3, we run an ensemble of 24 independent realisations for T=20 with a short transient $T_{\text{burn}}=2$. The sign-folded first and second moments 〈|u|(x)〉 and $\langle u^{2}\left(x\right)\rangle$ are compared against a fine-Δt SI-EM reference at $\Delta t=10^{-3}$ in Fig. 4. Our method at $\Delta t=2\;\times 10^{-2}$ agrees with the reference to within the Monte-Carlo noise of the ensemble (maximum relative difference 12.9% in the second moment, 7.4% in the first). Under the timing protocol specified below, the proposed method takes 1.11±0.01 s and the reference 8.40±0.06 s (mean ± s.d. over five repetitions), a wall-clock speedup of 7.5±0.1× at matched statistical accuracy. Fig. 3

### Stochastic burgers (stiff, chaotic)

**Fig. 5:**
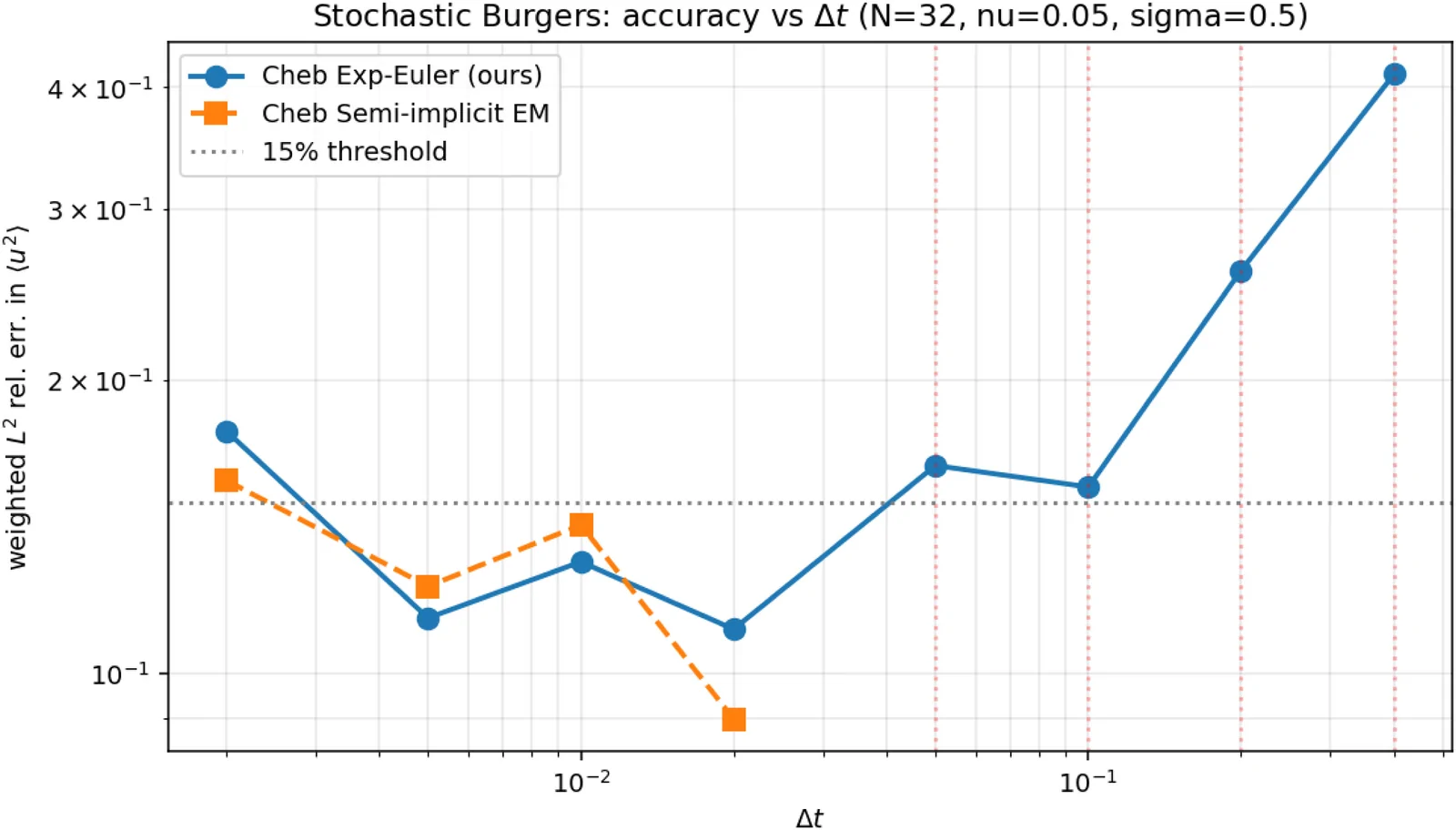
Stochastic Burgers time-step scan, N=32, ν=0.05, σ=0.5, T=16, 12 realisations per data point. Weighted $L^{2}$ relative error in $\langle u^{2}\rangle$ against a fine-Δt ($\Delta t=5\;\times 10^{-4}$) SI-EM reference. Vertical red dotted lines mark Δt values at which SI-EM blows up. Our exponential-Euler is stable and moderately accurate across the full range.

This case adds stiff, convection-dominated, chaotic dynamics and therefore stresses stability at large Δt. For $du=(\nu u_{xx}-uu_{x})\;dt+\sigma \;dW$ on [−1,1] with ν=0.05 and σ=0.5, we scan all four integrators across a range of time steps and record the largest step each can take before the solution blows up (Table 1). The proposed exponential-Euler remains stable across the entire range up to Δt=0.4 and stays within ∼19% of the fine-Δt reference up to Δt=0.1. Explicit Euler–Maruyama blows up for $\Delta t\geq 2\;\times 10^{-3}$, S-ROCK for Δt≥0.05, and SI-EM for Δt≥0.1. The proposed method therefore admits an 8× larger stable step than SI-EM, 20× larger than S-ROCK, and roughly 400× larger than explicit Euler–Maruyama on this problem (Fig. 5). We emphasise the correct interpretation of this result: within the region where two methods are *both* stable and accurate, their errors track each other closely (all integrators are weak order one, and the error there is dominated by the nonlinear term and the Monte-Carlo noise). The practical benefit of the proposed method on this stiff problem is therefore the substantially wider stable and usable time-step range — and the wall-clock saving that follows from it — rather than a higher accuracy at time steps where the competing methods also work.

**Table 1:** Largest stable time step (before blow-up) on stochastic Burgers (N=32, ν=0.05, σ=0.5), and the stable-step advantage of the proposed method.

**Integrator**	**Largest stable**Δt	**Ratio to proposed**
Proposed Cheb exp-Euler	≥0.4	—
Semi-implicit Euler–Maruyama	0.05	≥8×
S-ROCK (stabilized explicit)	0.02	≥20×
Explicit Euler–Maruyama	0.001	≥400×

### Ornstein–Uhlenbeck noise validation

**Fig. 6:**
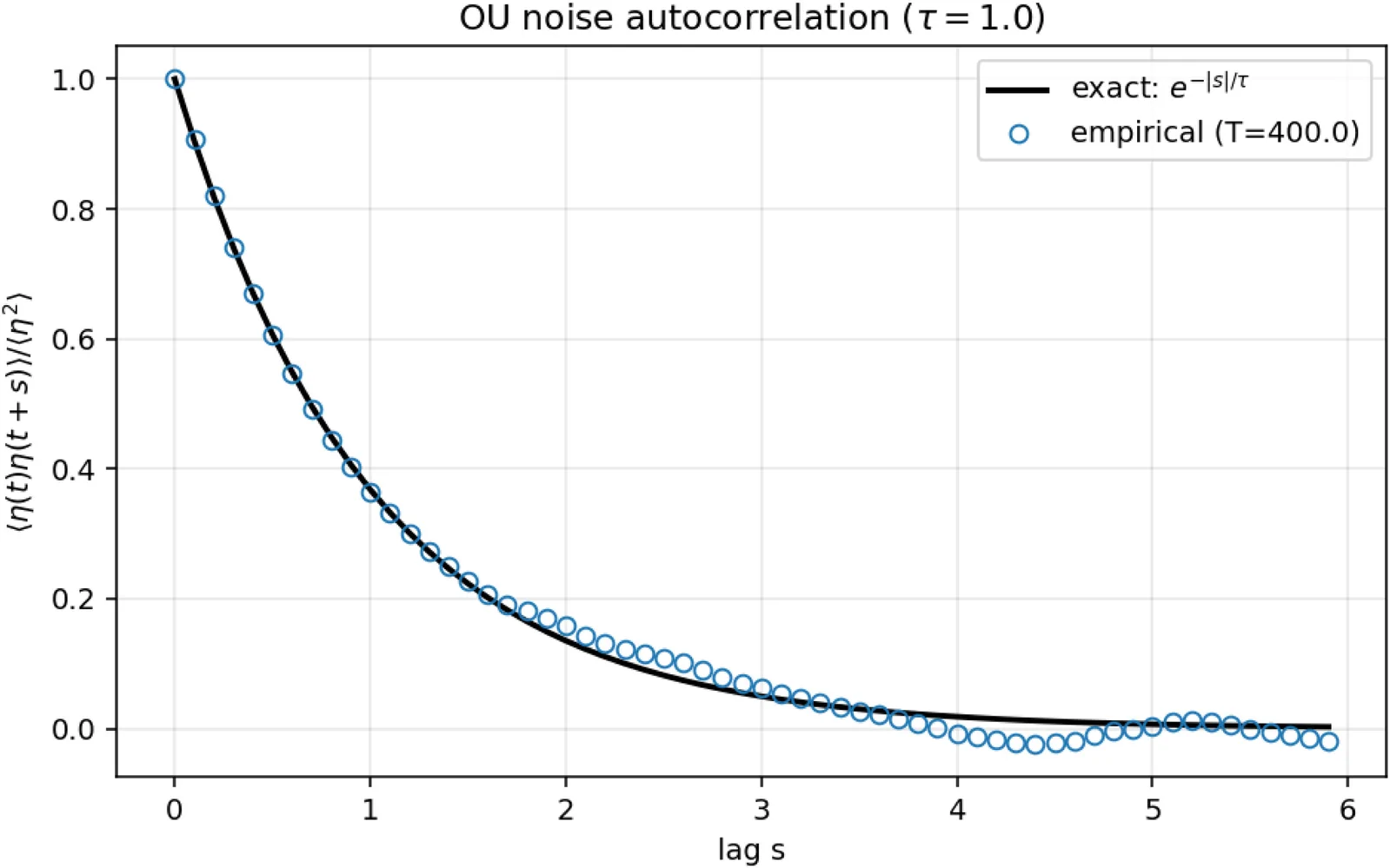
Ornstein–Uhlenbeck noise autocorrelation at an interior node, τ=1, T=400, Δt=0.01. Solid: exact $e^{-|s|/\tau }$. Circles: empirical estimate. Maximum absolute error across lags: 0.036.

This case tests temporally coloured noise, for which the correct autocorrelation and stationary variance must both be reproduced. We validate the OU integrator by checking these two defining properties. Fig. 6 shows the empirical autocorrelation of a single node of an OU field with τ=1, σ=1 over T=400, Δt=0.01, against the exact exponential $e^{-|s|/\tau }$; the maximum absolute error across lags in [0,6τ] is 0.036. The stationary variance check gives a maximum relative error of 4.8% at 3 standard errors across 20 ensemble runs - i.e. indistinguishable from Monte-Carlo noise, confirming the absence of bias. On the OU-driven linear SPDE $du=u_{xx}\;dt+\eta \left(x,t\right)\;dt$ with τ=0.5, σ=0.6, runs at Δt=0.02 and Δt=0.002 agree to within 8% over 25 realisations.

## Convergence study

We complement the pointwise validations above with a systematic convergence assessment in both space and time. For each we report a deterministic panel, in which no Monte-Carlo floor is present and the convergence rate is directly visible, together with its stochastic counterpart, in which the corresponding discretization error is compared against the statistical floor.

**Fig. 7:**
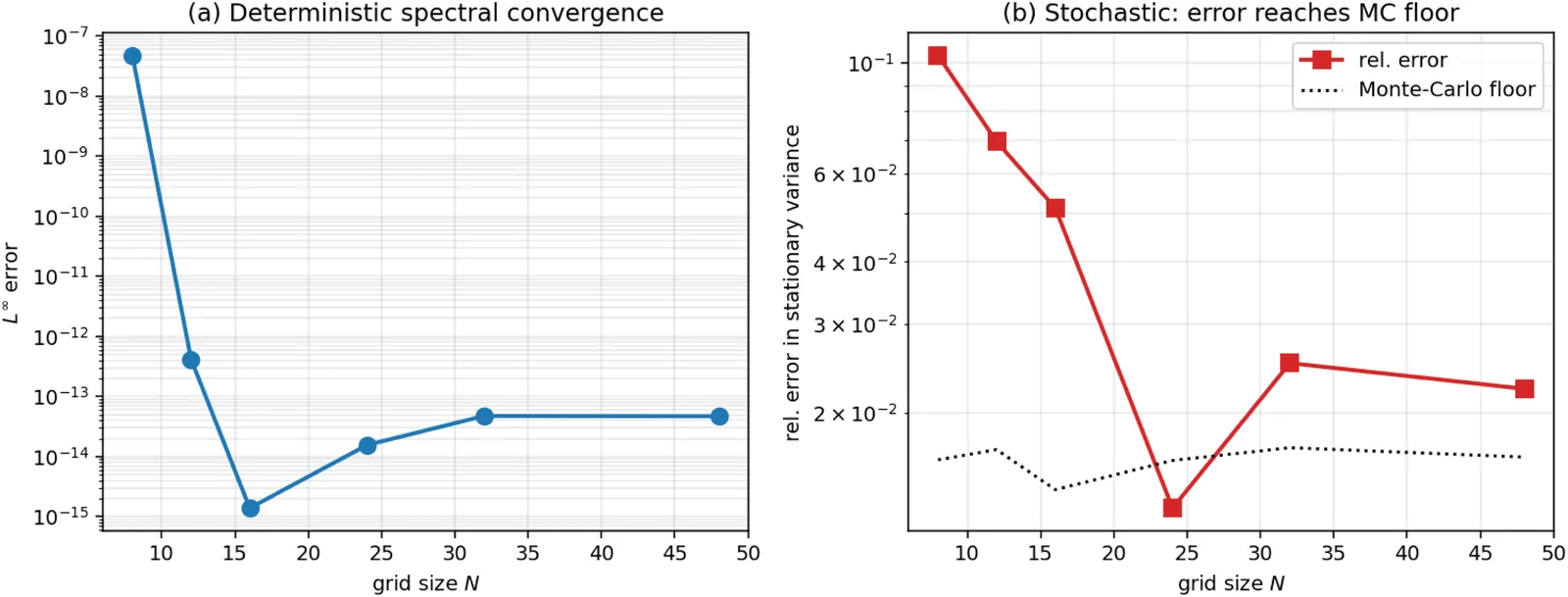
Spatial convergence. (a) Deterministic spectral convergence of the exponential propagator on the heat equation: $L^{\infty }$ error versus N, falling to machine precision by N=16. (b) Stochastic linear SPDE: relative error in the stationary variance versus N reaches the Monte-Carlo floor (dotted) near N≈24.

*Spatial.* On the deterministic heat problem the exponential propagator converges spectrally, with the $L^{\infty }$ error falling from $4.7\;\times 10^{-8}$ at N=8 to $1.4\;\times 10^{-15}$ at N=16 (Fig. 7a and the table above). For the stochastic linear SPDE the relative error in the stationary variance decreases from 10% at N=8 to the Monte-Carlo floor (≈1.6%) by N≈24 (Fig. 7b); beyond that, spatial error is below statistical error for the ensemble sizes used.

**Fig. 8:**
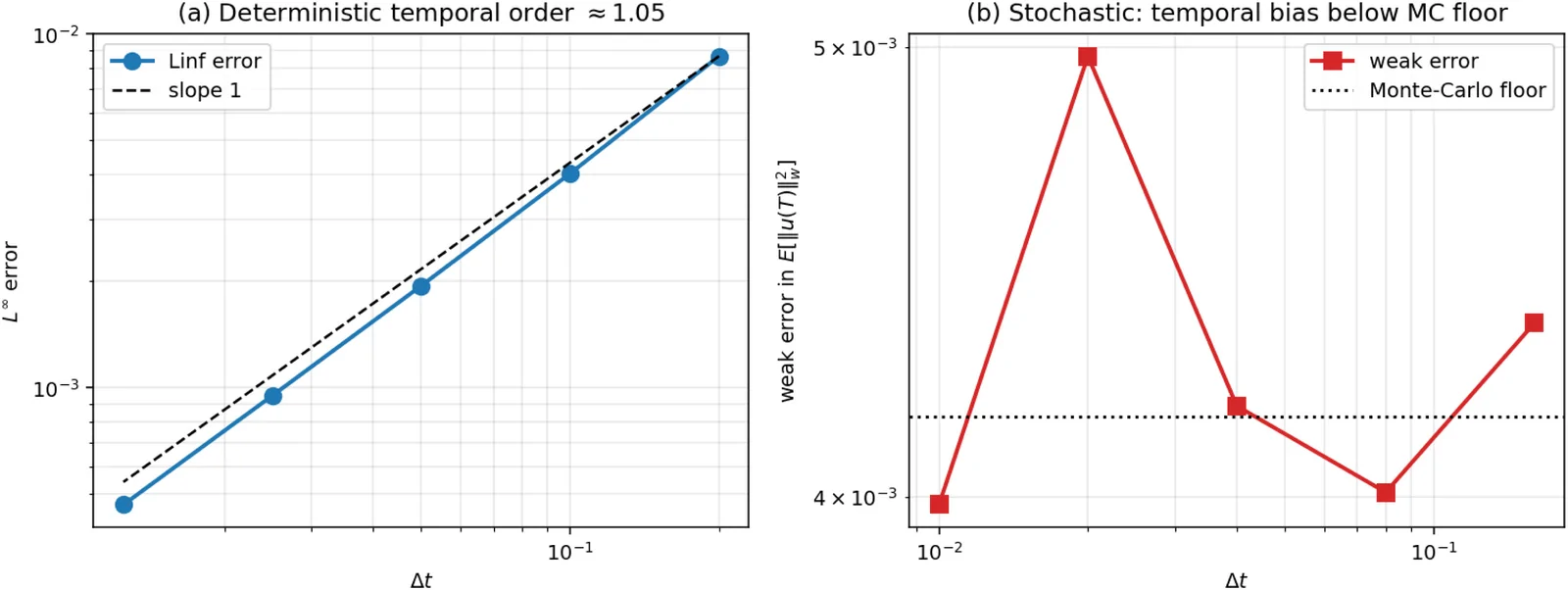
Temporal convergence. (a) Deterministic (σ=0) Allen–Cahn self-convergence: $L^{\infty }$ error versus Δt against a fine-Δt reference, with a fitted order of 1.05 (dashed guide: slope 1). (b) Stochastic Allen–Cahn: weak error in $E[{∥u\left(T\right)∥}_{w}^{2}]$ versus Δt stays at the Monte-Carlo floor (dotted), i.e. the temporal bias is below statistical error across the practical range.

*Temporal.* A self-convergence test on the deterministic (σ=0) Allen–Cahn problem, comparing each Δt against a fine-Δt reference, gives a fitted order of 1.05 (Fig. 8a), confirming the expected order one of the exponential-Euler treatment of the nonlinear term. For the stochastic problem the weak error in $E[{∥u\left(T\right)∥}_{w}^{2}]$ remains at the Monte-Carlo floor across Δt∈[0.01,0.16] (Fig. 8b): because the linear part is integrated exactly, the temporal bias of the scheme is negligible over the practical step range, consistent with (indeed sharper than) the weak-order-1 bound.

More generally, the scheme is expected to be weak order one in Δt — the order shared by SI-EM and S-ROCK — with the strong order limited by the temporal regularity of the additive stochastic convolution; the exact treatment of the linear part removes the linear contribution to the temporal error entirely, which is why the empirical temporal bias sits below the Monte-Carlo floor. A full theoretical strong/weak convergence proof for the dense, non-normal Chebyshev operator is beyond the scope of this contribution (see *Limitations*).

### Reproducibility and performance protocol

All timings reported above were obtained on a single machine: an Intel Core i7–​1165G7 @ 2.80 GHz (4 physical cores, 8 logical threads), 8.3 GB RAM, Windows 11 (build 10.0.26,200), running CPython 3.13.14 with NumPy 2.3.1 and SciPy 1.16.0 (scipy-openblas), with BLAS threading pinned to a single thread so that the small dense linear-algebra operations are timed deterministically. *Wall-clock* denotes the elapsed time.perf_counter time for the complete ensemble solve, excluding plotting and file I/O; each reported figure is the mean ± standard deviation over five independent timing repetitions (the first repetition, which additionally incurs interpreter and library warm-up, is included in the statistics). The *wall-clock advantage* of one method over another is the ratio of their wall-clock times at matched statistical accuracy. Every numerical value and figure in this article is regenerated by the scripts in the accompanying repository (folder revision/ for the added studies), each of which fixes its random seeds; a machine-readable summary of all numbers is provided there.

## Limitations

The method has the following limitations that users and reviewers should be aware of:

1. **Weak order 1 in** Δt**.** The stochastic exponential-Euler update is only weak-order 1, as are SI-EM and S-ROCK. For problems requiring strong-order or weak-order 2, a stochastic analogue of ETDRK4 would require evaluating the higher-order φ-functions $\varphi_{2},\varphi_{3}$ against the stochastic convolution, which we did not implement.
2. **No full convergence proof.** We provide an empirical convergence study (spectral in space, order one in time) and cite the expected weak/strong orders, but a rigorous strong/weak convergence analysis for the dense, strongly non-normal Chebyshev operator — including the dependence of the constants on the eigenvector conditioning of $L_{\text{int}}$ — is beyond the scope of this MethodsX contribution and is left to future work.
3. **Setup cost scales as** $O(N^{3})$**.** The initial eigendecomposition of $L_{\text{int}}$ and the Cholesky of the stochastic-convolution covariance are each $O(N^{3})$. For N>256, the setup dominates the first time step’s cost; after that, all work is $O(N^{2})$ per step. For very large N a Schur-based or Krylov-based stochastic convolution would be preferable.
4. **Non-normal eigenbasis.** For strongly non-normal $L_{\text{int}}$, the eigenvector matrix V is ill-conditioned, and the eigendecomposition-based stochastic convolution accumulates errors at large N. In our tests (N≤48), this was not observed; for larger N a fallback based on Schur–Parlett or a Lanczos-type method for f(A)b operations would be more robust.
5. **Additive noise only.** Multiplicative noise (N(u)dW) would require a Milstein-type correction that we did not implement. The present method is restricted to additive white or OU noise.
6. **1D only.** The 2D extension via tensor-product (Sylvester) or Krylov techniques is straightforward in principle but was not implemented here.

## Ethics statements

This work did not involve human subjects, animal experiments, or data collected from social media platforms.

## CRediT author statement

**Ronobir Chandra Sarker:** Conceptualization, Methodology, Software, Validation, Formal analysis, Investigation, Data curation, Writing — original draft, Writing — review and editing, Visualization. **Md. Shahidul Islam:** Conceptualization, Methodology, Writing — review and editing, Supervision. **Atiqur Rahman:** Methodology, Writing — review and editing, Supervision.

## Data availability

The Python implementation, including validation tests and demonstration scripts that reproduce every figure in this manuscript, is openly available under an MIT license at https://github.com/Ronobir1sarker/cheb-spde. No external datasets were used; all numerical results in the manuscript were generated by the provided code.

Supplementary material and additional information

The package cheb_spde contains four solver modules (chebyshev_grid.py, phi_functions.py, noise.py, spde_solver.py), three validation tests (test_deterministic.py, test_stationary_variance.py, test_ou_noise.py), four demonstration scripts (benchmark_linear.py, allen_cahn_demo.py, burgers_demo.py, burgers_dt_scan.py), and a README with installation and usage instructions. The additional studies introduced in this version are collected in the folder revision/: the two further comparators (comparators.py: explicit Euler–Maruyama and S-ROCK), the multi-method benchmark and confidence-interval figures (fig2_benchmark_multi.py, fig1_variance_errorbars.py), the spatial/temporal convergence study (convergence_study.py), and the reproducible timing and stability study (timing_study.py), together with a machine-readable results summary. All figures in this article can be regenerated in under ten minutes on a commodity laptop.

## Declaration of generative AI and AI-assisted technologies in the writing process

During the preparation of this work the authors used Anthropic’s Claude (an AI-based language model) in order to improve the readability and language of the manuscript and to assist with template formatting. After using this tool, the authors reviewed and edited the content as needed and take full responsibility for the content of the publication. AI tools were not used to generate scientific content, perform mathematical derivations, write code, or produce any of the validation results presented in this article; all such work is the authors’ own.

## Declaration of competing interest

The authors declare that they have no known competing financial interests or personal relationships that could have appeared to influence the work reported in this paper.

## Appendix Supplementary materials

Image, application 1
